# Dynamic Properties of Mineral-Based Cementitious Material-Stabilized Slurry Soil Under Vehicle Loading

**DOI:** 10.3390/ma18194539

**Published:** 2025-09-29

**Authors:** Zhenlong Sun, Yingying Zhao, Jun Luo, Fengxi Zhou, Xianzhang Ling, Yongbo Wang, Yaping Yang, Sanping Han

**Affiliations:** 1 School of Civil Engineering, Qingdao University of Technology, Qingdao 266033, China; szl0185@163.com; 2 China Institute of Water Resources and Hydropower Research, Beijing 100038, China; 3 School of Civil and Hydraulic Engineering, Lanzhou University of Technology, Lanzhou 730050, China; 4 School of Civil Engineering, Harbin Institute of Technology, Harbin 150090, China; 5 Taizhou Traffic Survey and Design Institute Co., Ltd., Taizhou 318000, China; 6 Inner Mongolia Urban Planning & Municipal Engineering Design Research Institute, Hohhot 010010, China; 7 China Railway 17th Bureau Group Co., Ltd., Taiyuan 030032, China

**Keywords:** cementitious materials, silt, dynamic triaxial test, dynamic shear modulus ratio, damping ratio

## Abstract

Sludge is a common engineering byproduct that poses environmental and land-use challenges when disposed of directly. Converting sludge into high-quality subgrade filling material through solidification is therefore of both engineering and ecological significance. In this study, dynamic triaxial tests were conducted on sludge soils stabilized with mineral-based cementitious binders to investigate the effects of binder content, loading frequency, and curing age on the backbone curve, dynamic shear modulus, maximum shear modulus, ultimate stress amplitude, shear modulus ratio, and damping ratio. Scanning electron microscopy (SEM) was further employed to examine the microstructural evolution of the stabilized soils. The results indicate that increasing binder content and curing age significantly enhance the dynamic shear modulus while reducing the damping ratio, and the modulus exhibits a frequency-dependent behavior within the tested loading range. The modified Hardin-Drnevich constitutive model was successfully applied to fit the experimental data, accurately characterizing the dynamic response of stabilized sludge soils and enabling the development of a normalized model for the dynamic shear modulus ratio. SEM observations confirm that hydration reactions between the binder and soil produce gel products that fill interparticle pores, leading to a denser structure and explaining the observed macroscopic improvements in mechanical behavior. Overall, this work elucidates the dynamic response mechanisms of sludge stabilized with mineral-based cementitious materials and provides theoretical and experimental support for its resource utilization in road engineering applications.

## 1. Introduction

With the rapid development of the social economy, the demand for road transportation continues to rise. The large-scale construction of road infrastructure has substantially increased the demand for natural soil materials. However, the large-scale extraction of natural soil is restricted by resource scarcity and ecological protection requirements. Meanwhile, the vast amounts of waste sludge generated in engineering and industrial activities exhibit unfavorable engineering properties, including high moisture content, large porosity, high compressibility, and low permeability, which make it unsuitable for direct application in construction projects [1,2,3,4]. Sludge can, however, be effectively stabilized to reduce its moisture content and improve its mechanical properties, thereby converting it into a material with favorable engineering performance for subgrade filling [5]. Owing to its low cost, high efficiency, effectiveness, and renewability, slurry soil stabilization technology has been widely adopted [6,7,8].

Although cement has been widely applied in engineering practice, its drawbacks—such as high consumption, low early strength, and susceptibility to cracking—have gradually become evident. These limitations have driven the search for more environmentally friendly and efficient alternative binders [9,10,11]. Solid waste materials, including phosphogypsum, fly ash, slag, and red mud, have been increasingly used as supplementary stabilizers to enhance the solidification performance and reduce cement consumption [12,13,14,15,16]. This approach not only mitigates the environmental pollution associated with solid waste disposal but also promotes its resource utilization.

Recent studies have applied solid waste materials as stabilizers for saline soils [17], loess [18], and slurry soils [19,20]. The mechanical behavior of these stabilized soils has been investigated using unconfined compressive strength tests [21] and triaxial shear tests [22], while their solidification mechanisms have been explored through microstructural analyses such as scanning electron microscopy (SEM) [23]. For instance, Zhou Zhen et al. [24] employed recycled construction aggregates and mine tailings powder combined with cement to stabilize slurry soils, determining the optimal mix ratio (5% cement, 7.8% blast furnace slag powder, and 13.8% recycled aggregates) using response surface methodology. The stabilized soil achieved an unconfined compressive strength of ≥2.5 MPa after 7 days of curing. Similarly, Ye et al. [25] developed a GS stabilizer composed of slag, steel slag, fly ash, desulfurization gypsum, and cement. Their unconfined compressive strength and SEM results demonstrated that GS exhibited superior solidification performance compared to cement alone. Li Xuehe et al. [26] used a mixture of slag, steel slag, desulfurization gypsum, and ordinary Portland cement to solidify Huanghe alluvial silt. They observed a synergistic hydration effect during solidification, with early ettringite formation followed by the later development of C–S–H gel. This process produced a dense composite structure, significantly improving the strength of the stabilized silt.

Currently, research on the solidification of soils with solid waste materials has primarily focused on static properties, while studies addressing dynamic behavior remain relatively limited. In engineering practice, however, subgrades are subjected not only to static loads but also to dynamic loads arising from traffic [27] and seismic actions [28,29]. By contrast, the dynamics of road structures, modeled as multilayer transversely isotropic media, have been extensively investigated and successfully applied in engineering practice [30,31,32], particularly under vehicle and seismic loading. These studies provide valuable insights into the dynamic response of layered geotechnical systems. Nevertheless, despite such progress, systematic investigations into the dynamic characteristics of sludge soils stabilized with mineral-based cementitious materials remain insufficient. Therefore, this study aims to elucidate the dynamic response mechanisms of stabilized sludge soils through dynamic triaxial testing and SEM analyses, thereby providing theoretical support for their practical application in transportation infrastructure.

## 2. Experimental Materials and Design

### 2.1. Experimental Materials

**Figure 1:**
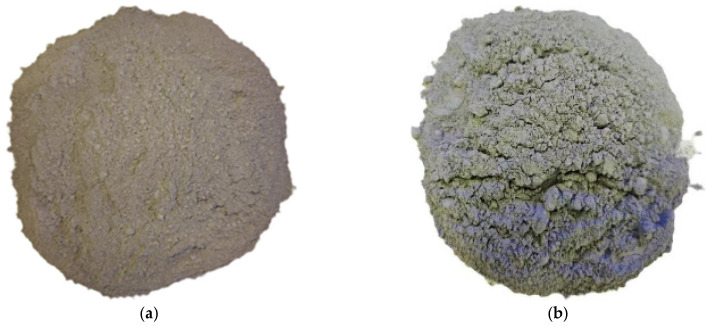
Experimental Materials. (**a**) silt; (**b**) Mineral-based Cementitious Material.

**Figure 2:**
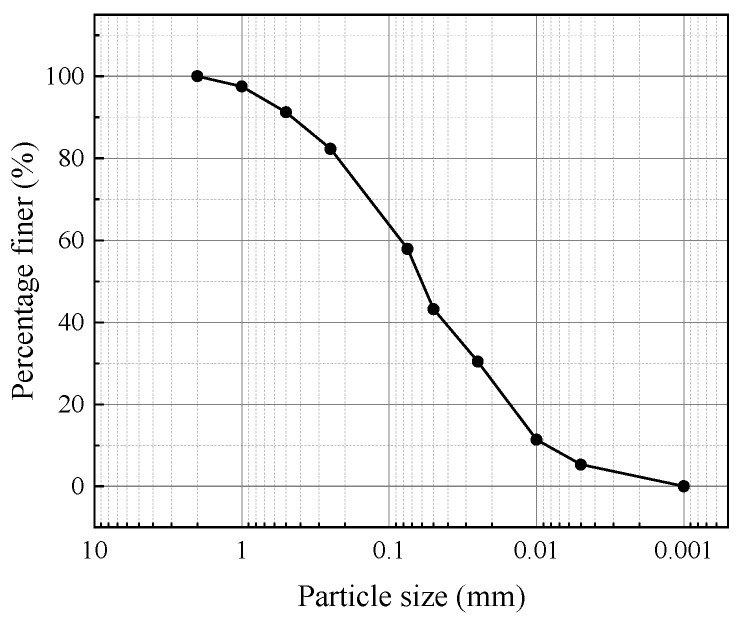
Particle Size Distribution Curve of Slurry Soil.

**Figure 3:**
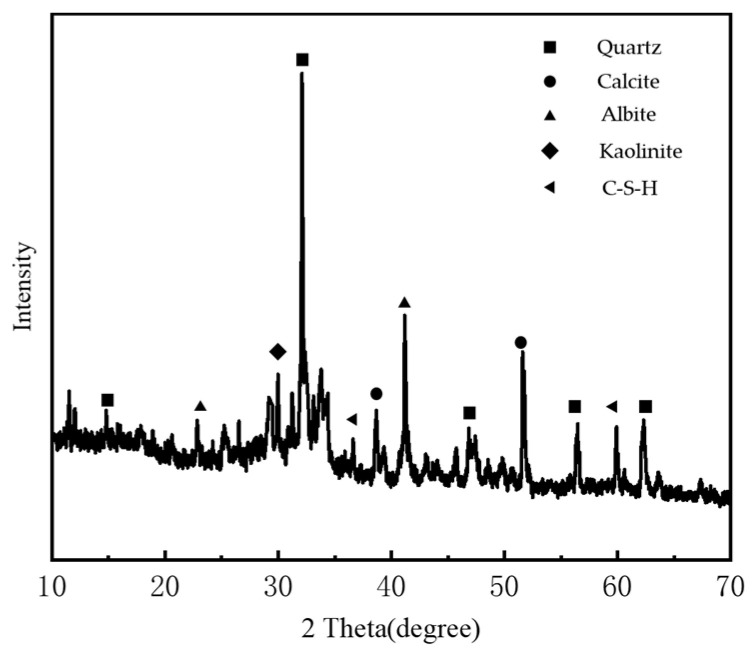
XRD pattern.

The soil samples were collected from the engineering site, with a natural moisture content of 50.6%. The samples were oven-dried at 105 °C to a constant weight, then ground and sieved through a 2 mm mesh. The processed soil (representative of the collected samples) is shown in Figure 1a. The main mineral constituents are quartz, kaolinite, illite, and minor phases. The maximum dry density of the soil was determined to be 1.69 g/cm^3^, and the optimum moisture content was 17.0%, as obtained from standard compaction tests [33]. The basic physical properties of the soil are summarized in Table 1, and the particle size distribution curve is shown in Figure 2. The mineral-based cementitious material used in this study was supplied by Qingdao Panyao New Materials Engineering Research Institute Co., Ltd., located in Qingdao, Shandong, China. Its primary chemical composition is listed in Table 2. In addition, X-ray diffraction (XRD) analysis (Rigaku Corporation, Tokyo, Japan) was conducted on the specimens, and the corresponding diffraction patterns are presented in Figure 3.

### 2.2. Sample Preparation

To minimize experimental errors, a calculated amount of distilled water was added to the soil samples. After thorough mixing, the samples were sealed in plastic bags and allowed to equilibrate for 24 h to ensure uniform moisture distribution. The mineral-based cementitious material was then weighed according to the designed mix ratio and uniformly blended with the preconditioned soil. Standard cylindrical specimens were prepared using the layer-wise compaction method. Each specimen consisted of five layers, with the height of each layer controlled at 28 mm. After compaction, the surface of each layer was carefully leveled to improve interlayer bonding. Upon demolding, the specimens were transferred to a standard curing chamber maintained at a temperature of 20 ± 2 °C and a relative humidity of 95%. The samples were cured under these conditions until the specified curing age was reached.

### 2.3. Experimental Methods

#### 2.3.1. Dynamic Triaxial Test

**Figure 4:**
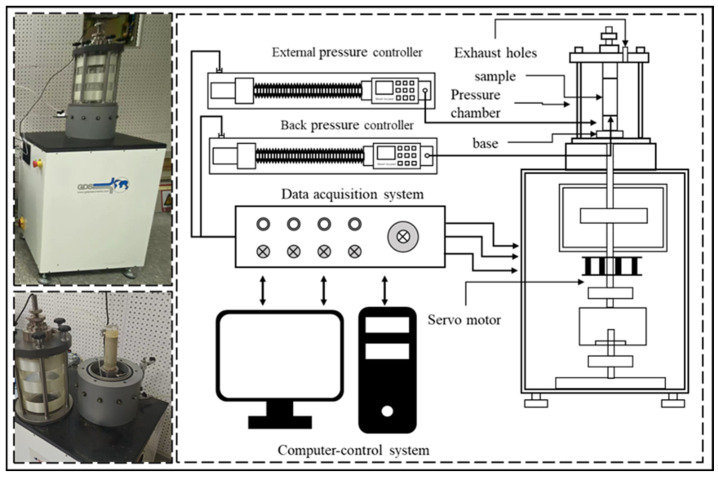
GDS Dynamic Triaxial System.

The dynamic triaxial tests were performed using a GDS Dynamic Triaxial Testing System(Earth Products China Limited, Hong Kong, China) (Figure 4). The apparatus comprises a pressure chamber, dynamic loading device, volume controller, and data acquisition unit. The main technical specifications are as follows: maximum axial load of 10 kN, maximum axial displacement of 200 mm, loading frequency range of 0–5 Hz, confining pressure range of 0–2 MPa, and specimen dimensions of 70 mm in diameter and 140 mm in height. The system consists of three functional modules: standard loading, dynamic loading, and advanced loading. In this study, the dynamic loading module was employed to apply cyclic loads to the specimens, enabling the evaluation of their dynamic properties, particularly the dynamic shear modulus and damping ratio.

This study investigates the effects of mineral-based cementitious material content (c), loading frequency (f), and curing age (t) on the dynamic properties of stabilized soils. The experimental design is summarized in Table 3. Consolidated undrained triaxial tests were performed on unsaturated specimens. Each specimen was first subjected to isotropic consolidation under the prescribed confining pressure and axial stress, with the consolidation criterion defined as an axial strain rate lower than 0.01 mm/h ($\sigma_{3}$ = 60 kPa).

Subsequently, cyclic loading was applied using a sine wave to simulate traffic loads [34]. A unilateral loading scheme was adopted, in which the consolidated axial stress served as the minimum stress, thereby approximating the time–load relationship of subgrade soils under traffic loading [35]. According to the highway traffic load parameters reported by Luo Jin [36], the loading frequencies were set to 1 Hz, 2 Hz, and 3 Hz. The loading protocol employed an initial dynamic stress amplitude of 0 kPa, followed by stress increments of 40 kPa per stage, with 13 load cycles in each stage, as illustrated in Figure 4.

Specimens were considered to have failed when they could no longer withstand additional loading, at which point the test was terminated. Using this methodology, the dynamic properties of sludge soil under different cementitious material contents, loading frequencies, and curing ages were systematically investigated. The results provide both theoretical insights and experimental data to support engineering applications.

#### 2.3.2. Scanning Electron Microscopy (SEM) Test

**Figure 5:**
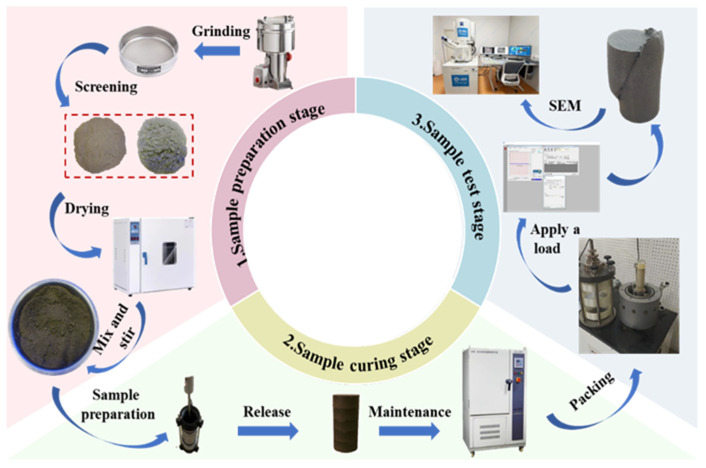
Testing Procedure.

The scanning electron microscopy (SEM) tests were conducted using a microscope at the School of Environmental and Municipal Engineering, Qingdao University of Technology. Representative samples were taken from the central portions of the triaxial specimens. To ensure complete dehydration, the samples were oven-dried at 105 °C for 24 h. The dried specimens were then mounted on the sample stage, subjected to vacuum freeze-drying, and subsequently coated with a thin layer of gold. SEM analyses were then performed to examine the microstructural characteristics of the stabilized soils. The experimental procedure is illustrated in Figure 5.

## 3. Data Processing Methods and Constitutive Model

The GDS Dynamic Triaxial Automatic Data Acquisition System can measure the axial displacement, axial force, lateral displacement, and lateral force at each load level. Using these measurements, the dynamic shear stress amplitude and dynamic shear strain at each load level during the loading process can be calculated.

(1) $$\gamma_{d}=\varepsilon_{d}\left(1+\mu \right)$$

(2) $$\tau_{d}=\frac{\sigma_{d}}{2}$$

In the above equation, $\gamma_{d}$ denotes the dynamic shear strain; $\varepsilon_{d}$ denotes the (axial) strain; μ denotes Poisson’s ratio; $\tau_{d}$ denotes the dynamic shear stress; and $\sigma_{d}$ denotes the (deviatoric) stress.

**Figure 6:**
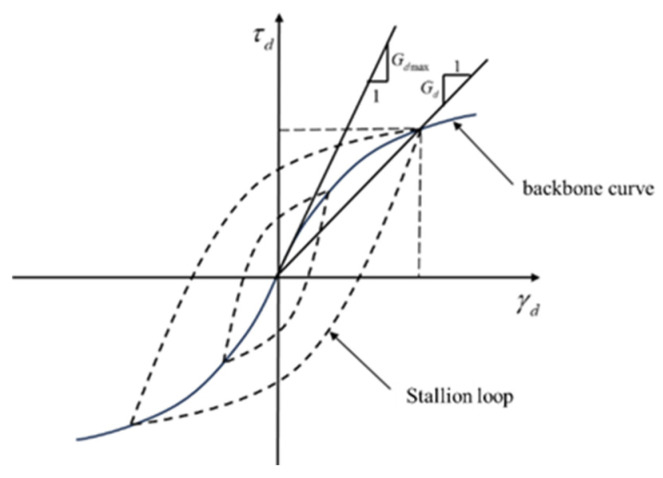
Stress–Strain Relationship of Soil under Cyclic Loading.

The stress–strain relationship of the solidified slurry soil in this study was depicted using the hyperbolic curve provided by Kondner, Hardin, and Drnevich [37] (as shown in Figure 6):

(3) $$\tau_{d}=\frac{\gamma_{d}}{a+b\gamma_{d}}$$

(4) $$1/G_{d}=a+b\gamma_{d}$$

In the above equation, a and b are experimental parameters of the soil, where a > 0 and b > 0. Clearly, 1/a represents the slope of the backbone curve at the origin, and 1/b is the intercept of the horizontal asymptote of the backbone curve on the vertical axis.

The dynamic shear modulus of the soil can be obtained from the backbone curve, and its expression is given by [38]:

(5) $$G_{d}=\frac{\tau_{d\max}-\tau_{d\min}}{\gamma_{d\max}-\gamma_{d\min}}$$

In the above equation, $\tau_{d\max}$ and $\tau_{d\min}$ represent the maximum and minimum dynamic shear stress, respectively, under the same cyclic load, while $\varepsilon_{d\max}$ and $\varepsilon_{d\min}$ are the corresponding maximum and minimum dynamic.

(6) $$\lambda =\frac{1}{4\pi }\cdot \frac{W_{T}}{W}=\frac{1}{4\pi }\cdot \frac{S}{S_{T}}$$

In the above equation, $W_{T}$ represents the energy dissipated by the soil under a single dynamic load cycle, and W is the total energy stored by the soil under the same load. S denotes the area of the hysteresis loop, and $S_{T}$ is the area of the triangle formed by the projection of the maximum amplitude point on the horizontal axis and the origin.

## 4. Experimental Results and Analysis

### 4.1. Backbone Curve Analysis

**Figure 7:**
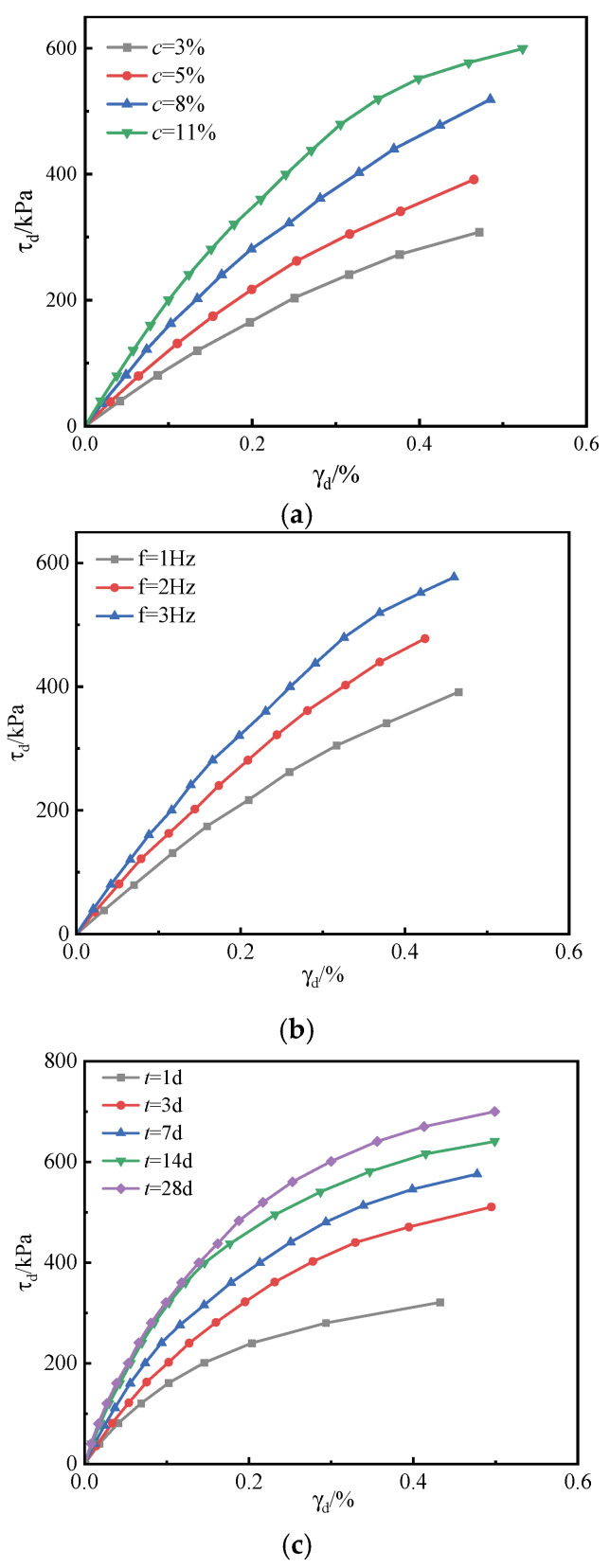
Backbone curve of mineral-based cementitious material solidification sludge. (**a**) Mineral-based Cementitious Material. (**b**) loading frequency. (**c**) curing age.

Figure 7 presents the backbone curves of slurry soils stabilized with cementitious materials under different experimental conditions. These curves were derived by connecting the peak points of the dynamic shear stress-dynamic shear strain hysteresis loops at the 12th cycle of each loading stage. The backbone curves capture the evolution of dynamic shear stress-strain behavior of the stabilized soils subjected to cyclic loading [39].

The influence of binder content and curing age on the backbone curves of stabilized soils is illustrated in Figure 7a,c. With increasing binder dosage and curing period, the dynamic shear modulus of the stabilized soils exhibits a marked improvement, demonstrating that both parameters effectively enhance soil stiffness. This enhancement arises from the progressive filling of soil pores by hydration products, which increases soil compaction and strengthens the skeletal framework. As a result, the overall structural integrity is improved, and the soils display greater resistance to deformation under identical dynamic shear stress conditions.

As illustrated in Figure 7b, loading frequency exerts a pronounced influence on the backbone curve. With increasing frequency, the curve shifts upward, indicating that, under identical dynamic shear stress, the stabilized soil develops substantially greater shear strains at lower frequencies compared with higher frequencies. This phenomenon can be attributed to the accelerated energy transmission at higher frequencies, which reduces the relative displacement between particles and, consequently, suppresses dynamic shear strain.

### 4.2. Analysis of Dynamic Shear Modulus

**Figure 8:**
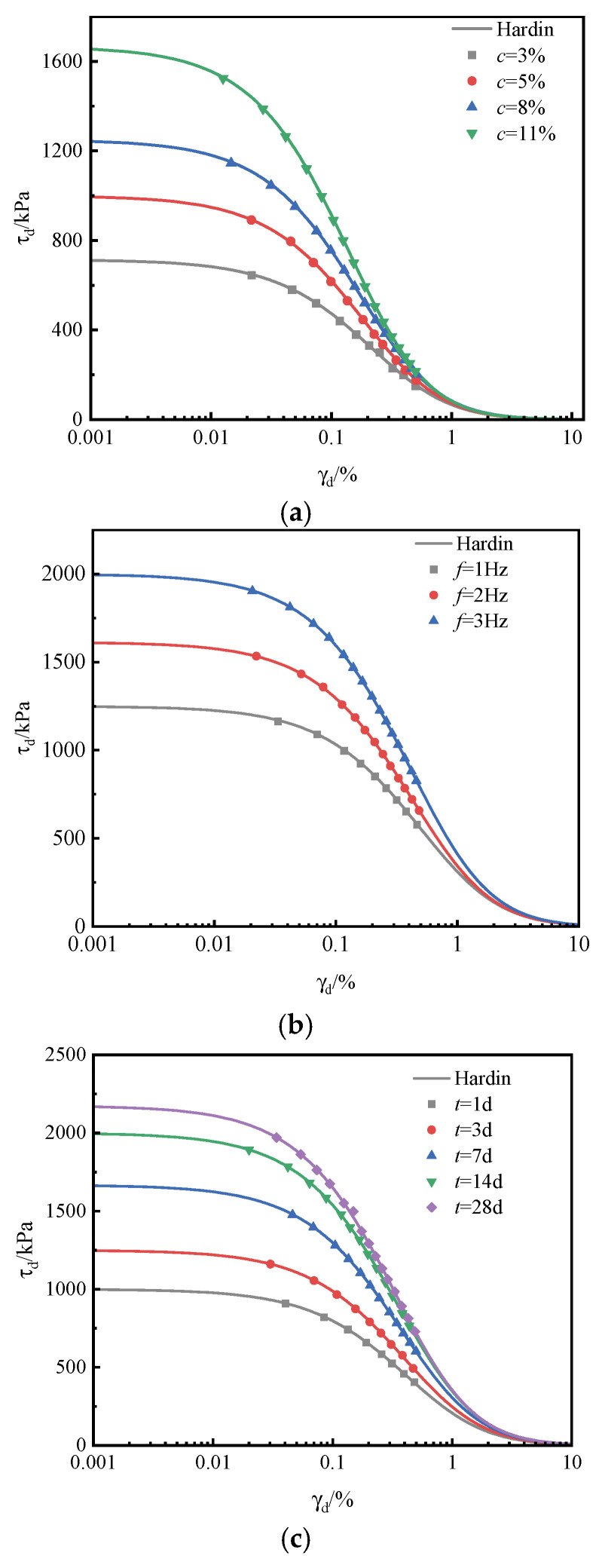
The relationship curve between dynamic shear modulus and dynamic strain under different test conditions. (**a**) Content of mineral-based cementing materials. (**b**) Loading frequency. (**c**) Curing age.

Figure 8 presents the relationship between the dynamic shear modulus and dynamic shear strain of cemented slurry soil under various experimental conditions, with the curves obtained by fitting the experimental data using the Hardin model. The results demonstrate that the dynamic shear modulus–strain curves exhibit consistent patterns across different conditions. When the dynamic shear strain is below 0.01%, the modulus decreases gradually; however, as the strain increases, the rate of decay accelerates markedly. Once the dynamic shear strain approaches 1%, the curve tends to level off, highlighting the nonlinear response of the cemented slurry soil. This behavior is attributed to the strain-softening phenomenon observed in the backbone curve as dynamic shear stress increases.

Figure 8a,c show that increasing cementitious material content and curing age shifts the dynamic shear modulus curve upward. In other words, for the same dynamic shear strain, both parameters exhibit a positive correlation with the modulus. This enhancement is attributed to the greater formation of hydration products, which densify the soil structure, increase stiffness, and thereby improve the soil’s resistance to deformation under cyclic loading.

Figure 8b demonstrates that increasing loading frequency shifts the dynamic shear modulus curve upward. In other words, for the same dynamic shear strain, the modulus of the stabilized soil increases with frequency. This behavior arises because high-frequency vibrations enhance interparticle friction among hydration products in the silt soil, thereby amplifying molecular damping effects. As a result, particle displacement is more strongly constrained, ultimately improving the shear resistance of the soil. In Figure 8b, it can be observed that, as the frequency increases, the dynamic shear modulus curve of the stabilized soil shifts upward. In other words, under the same dynamic shear strain conditions, the dynamic shear modulus of the soil increases with frequency. This is because high-frequency vibrations enhance the inter-particle friction of the hydration products of the cementing materials in the silt soil. The molecular damping effect increases accordingly, which imposes a stronger constraint on the displacement of soil particles, ultimately improving the shear strength of the soil.

### 4.3. Maximum Shear Modulus and Ultimate Stress Amplitude

**Figure 9:**
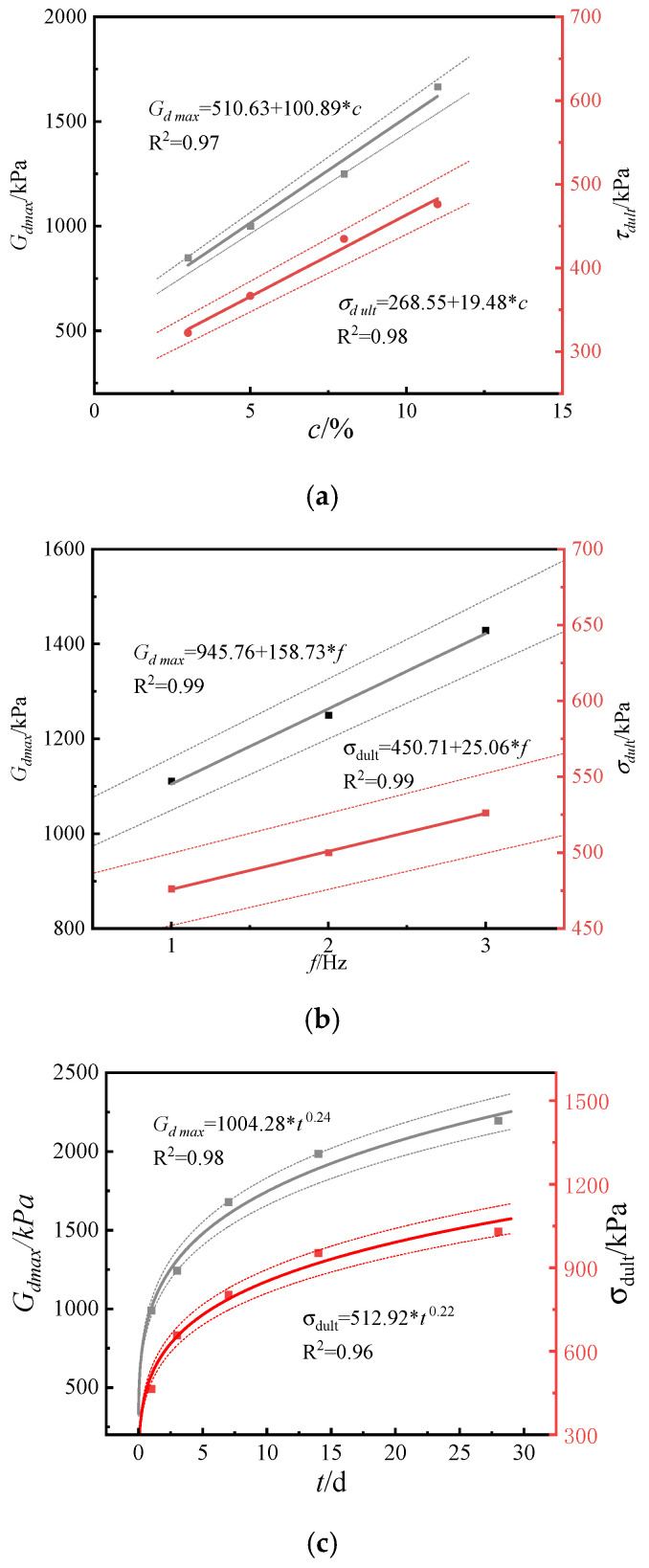
The relationship between the maximum shear modulus and the final stress amplitude under different test conditions. (**a**) Content of mineral-based cementing materials. (**b**) Loading frequency. (**c**) Curing age.

Figure 9 presents the relationship between the maximum shear modulus and the ultimate shear stress under varying experimental conditions. Both parameters exhibit strong correlations with binder content, loading frequency, and curing age. Specifically, binder content (c), frequency (f), and curing age (t) show positive correlations with the maximum shear modulus and ultimate stress amplitude. Moreover, binder content (c) and frequency (f) demonstrate a distinct linear relationship with both parameters, which can be expressed by the following equation (three data points are not representative):

(7) $$G_{d\max}=m+nc\left(f\right)$$

(8) $$\tau_{dult}=u+vc\left(f\right)$$

In the above equation, m, n, u, and v are the fitting parameters.

The curing age (t) exhibits a good power function relationship with both the maximum shear modulus and the ultimate stress amplitude. This relationship can be expressed by the following equation:

(9) $$G_{d\max}=pt^{q}$$

(10) $$\tau_{dult}=xt^{y}$$

In the above equation, p, q, x, and y are the fitting parameters.

### 4.4. Empirical Model for Normalized Dynamic Shear Modulus

The modified Hardin–Drnevich model (11) was used to perform a normalized regression analysis on the $G_{d}/G_{d\max}-\gamma_{d}$ relationship curves of cementing material-stabilized silt under different experimental conditions, resulting in the following empirical model for the normalized dynamic shear modulus of stabilized silt:

(11) $$\frac{G_{d}}{G_{d\max}}=\frac{1}{1+{\left(\gamma_{d}/\gamma_{r}\right)}^{\alpha }}$$

In the above equation, $G_{d\max}$ represents the initial maximum shear modulus; $\gamma_{d}$ is the shear strain; $\gamma_{r}$ is the reference shear strain, the unit is %; and α is the fitting coefficient.

**Figure 10:**
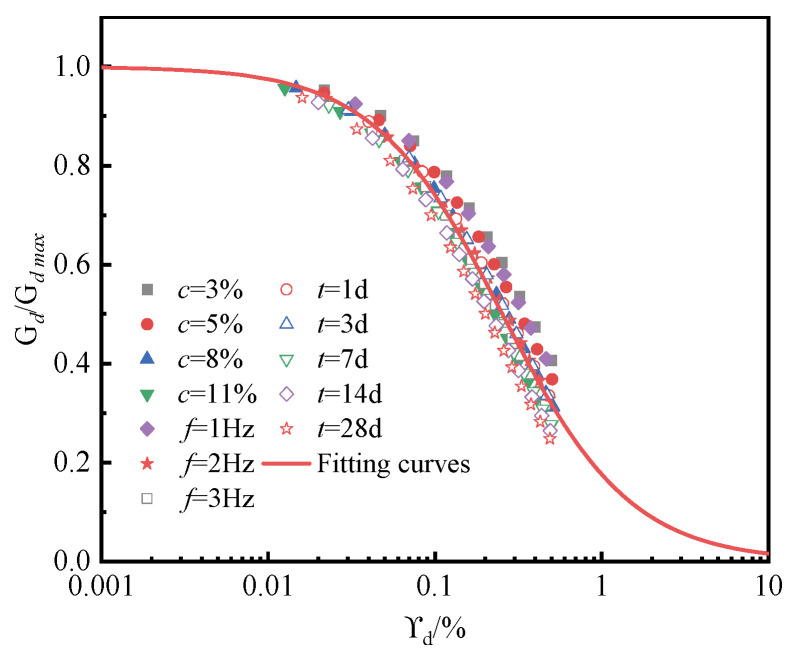
Normalized dynamic shear modulus ratio vs. dynamic shear strain curve.

The variation in the dynamic shear modulus ratio of stabilized silt under different experimental conditions is shown in Figure 10. It can be observed that at low dynamic shear strains, the dynamic shear modulus ratio of the soil decreases slowly. However, as the dynamic shear strain increases, the dynamic shear modulus ratio of the soil drops sharply. Moreover, the $G_{d}/G_{d\max}-\gamma_{d}$ relationship curves under different experimental conditions are distributed within a narrow region. The $G_{d}/G_{d\max}-\gamma_{d}$ relationship curves of cementing material-stabilized silt under different experimental conditions were normalized and fitted using Equation (11). The results indicate that the fitting parameters are $\gamma_{r}$ = 0.25 and α = 1.12, with a correlation coefficient R^2^ = 0.96, demonstrating that the model can effectively describe the variation in the dynamic shear modulus of stabilized silt under different experimental conditions. This indicates that the model can effectively describe the variation in the dynamic shear modulus of cement-stabilized silt under the different experimental conditions considered in this study.

### 4.5. Damping Ratio

**Figure 11:**
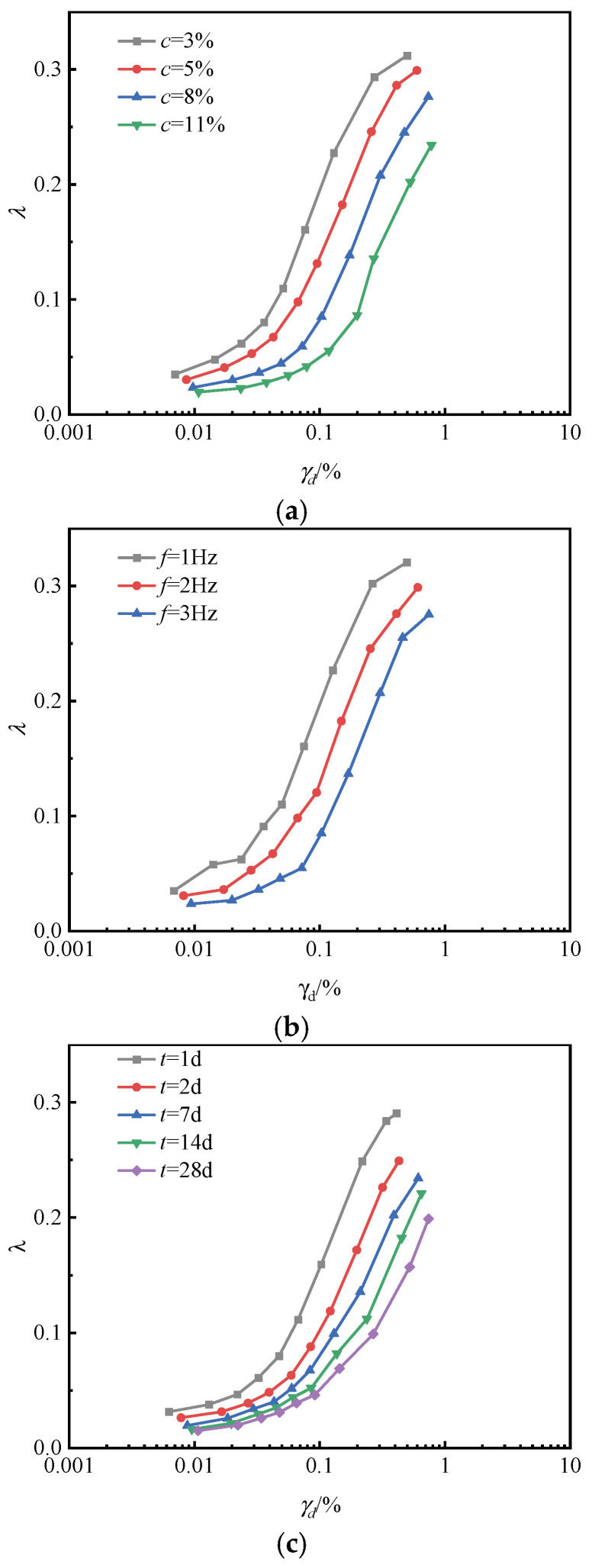
The relationship curve of damping ratio under different test conditions. (**a**) Content of mineral-based cementing materials. (**b**) Loading frequency. (**c**) Curing age.

Figure 11 illustrates the relationship between the damping ratio (λ) and dynamic shear strain of stabilized silt under different binder contents, loading frequencies, and curing ages. Overall, the damping ratio (λ) increases with dynamic shear strain. At the initial loading stage, when the dynamic shear strain is relatively small, the damping ratio rises slowly because the internal structure of the soil remains largely intact. As the dynamic shear strain continues to increase, however, progressive structural degradation occurs, resulting in greater internal frictional energy dissipation and a rapid increase in the damping ratio.

From Figure 11a,c, it can be seen that with an increase in the cementing material content and curing age, the $\lambda -\gamma_{d}$ relationship curve shifts downward. In other words, under the same dynamic shear strain, the damping ratio becomes smaller. This suggests that as the cementing material content and curing age increase, the amount of hydration products also increases, enhancing the skeleton effect, which results in a denser soil structure. Consequently, the energy dissipation is reduced, leading to a smaller damping ratio.

From Figure 11b, it can be seen that with increasing loading frequency, the curve shifts downward, indicating that the damping ratio of the soil decreases under the same shear strain conditions. This behavior can be attributed to high-frequency loading, which suppresses the full development of dynamic shear strain in the soil samples and thereby reduces internal energy dissipation. Consequently, the damping ratio decreases.

### 4.6. Microscopic Structure Analysis

#### 4.6.1. Qualitative Analysis

**Figure 12:**
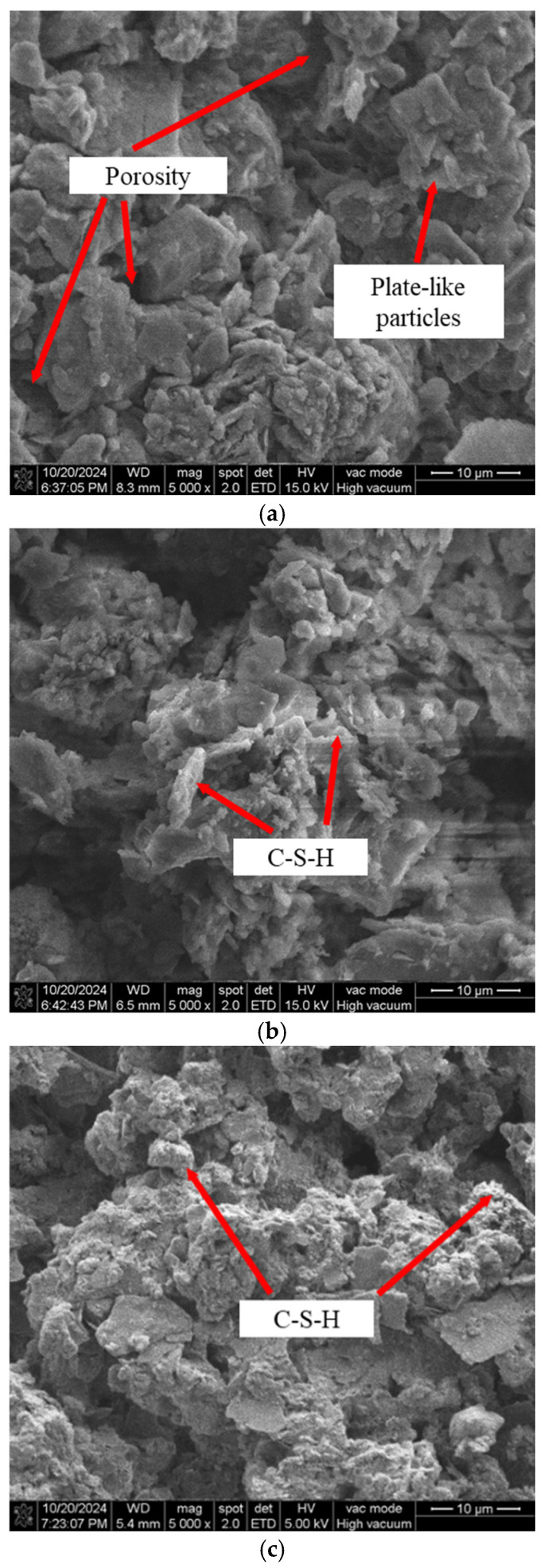
Scanning Electron Microscopy (SEM) Images of Microstructure. (**a**) Untreated soil.; (**b**) c = 3%. (**c**) c = 5%.

Figure 12 presents scanning electron microscopy (SEM) images of the silt at different cementing material contents (magnification: 5.0 k). In the untreated soil, particles exhibit a laminated structure with little to no bonding between them. As the cementing material content increases, the soil particles become more densely packed and the pore spaces decrease. This is attributed to the chemical reactions of CaO, SiO_2_, and Na_2_O in the cementing material, which generate calcium silicate hydrate (C-S-H) gel. A higher cementing material content accelerates these reactions, resulting in increased C-S-H production that fills the soil pores, enhances particle cohesion, and improves soil compactness. These microstructural changes modify particle interactions and strain transmission pathways, thereby influencing the dynamic response of the soil.

#### 4.6.2. Quantitative Analysis

The microstructural pore parameters of stabilized silt with varying cementitious material contents were quantitatively analyzed using ImageJ software. The analysis focused on pore size, morphology, and spatial distribution characteristics under different binder contents.

**Figure 13:**
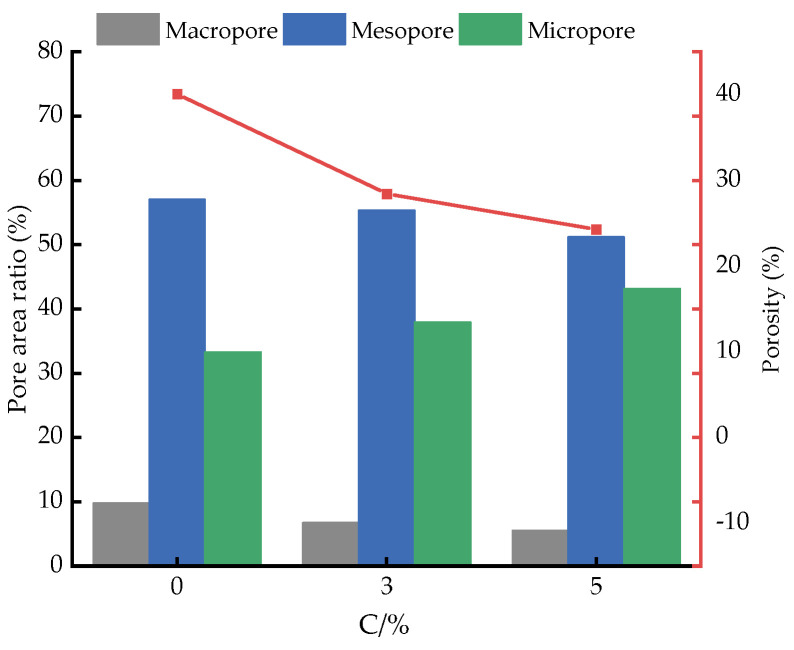
Distribution of soil cross-sectional porosity.

Figure 13 illustrates the variation in pore size distribution of soils with different contents of mineral-based cementitious material. In this study, soil pores were classified into macropores (d > 50 μm), mesopores (2 μm < d < 50 μm), and micropores (d < 2 μm). A bar chart was generated to present the relationship between pore size proportions and cementitious material content (C). As shown, with increasing cementitious material content, the internal pores of the soil are progressively filled due to continuous hydration reactions. Consequently, both macropores and mesopores exhibit a decreasing trend, whereas micropores show an overall increase.

**Figure 14:**
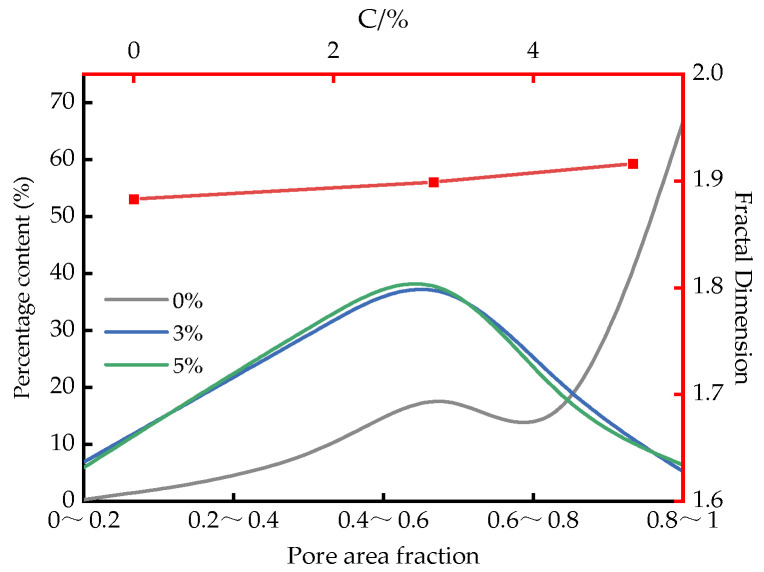
Morphological changes in particles under different contents of mineral-based cementitious materials.

The abundance of structural elements is defined as the ratio of the short axis to the long axis of a structural unit within the measurement window, ranging from 0 to 1, thereby reflecting the geometric characteristics of the structural elements. The variation in abundance under different cementitious material contents is shown in Figure 14. As observed, the abundance of the specimens is primarily distributed between 0.4 and 0.8, indicating that elongated structural elements are relatively rare, while most elements are equiaxed, with the soil matrix being dominated by ellipsoidal structures of varying degrees. Moreover, with increasing cementitious material content, the proportion of elements with abundance values in the range of 0.4–0.8 gradually increases, suggesting a progressive evolution of particle shapes toward ellipsoidal and near-spherical forms.

The fractal dimension reflects the uniformity and complexity of pore distribution. As shown in the figure, the fractal dimension remains relatively stable, ranging from 1.90 to 1.95. This suggests that the incorporation of cementitious material increases the surface roughness of soil particles. This effect results from the hydration reactions between the cementitious material and silt, which generate a large quantity of cementitious products and thereby enhance the complexity of the internal particle spatial structure. Such microstructural modifications influence particle interactions and strain transmission, ultimately affecting the dynamic response of the soil.

## 5. Discussion

### 5.1. Comparison with Previous Studies

Compared with existing studies that mainly focus on the static mechanical properties of stabilized soils, our results show significant improvements in the dynamic shear modulus and damping ratio of sludge soil stabilized with mineral-based cementitious materials. These findings are consistent with the general trends reported for cement-stabilized soils, while emphasizing the unique contributions of mineral-based binders under dynamic loading.

### 5.2. Mechanism Interpretation

SEM analysis revealed that hydration products (e.g., C–S–H gel, ettringite) fill interparticle voids and refine the microstructure. Such densification explains the observed improvements in shear modulus and the evolution of damping ratio with curing age and binder content, thereby linking microscopic mechanisms to macroscopic dynamic behavior.

### 5.3. Engineering Implications

The enhanced stiffness and favorable damping properties suggest that stabilized sludge soil is suitable for subgrade applications in transportation infrastructure. Its ability to resist traffic- and earthquake-induced dynamic loads also promotes sustainable utilization of waste sludge, reducing reliance on natural soils.

### 5.4. Limitations and Future Work

This study is limited by the narrow loading frequency range and single soil source. Future work should incorporate frequency-dependent variables into constitutive models, extend the frequency range, and validate laboratory findings through large-scale field tests to ensure broader applicability.

## 6. Conclusions

This study investigated the dynamic behavior of sludge soil stabilized with mineral-based cementitious materials through dynamic triaxial, SEM, and XRD tests. The main scientific novelties and contributions are as follows:

(1) Unlike previous studies that mainly focused on static properties, this work systematically elucidates the dynamic response of stabilized sludge under varying binder contents, curing ages, and loading frequencies, thereby bridging a critical research gap.
(2) A normalized constitutive model for the dynamic shear modulus ratio was proposed and validated, achieving a correlation coefficient of 0.96. This provides a novel predictive tool for describing the modulus–strain evolution of stabilized sludge soils with high accuracy.
(3) By integrating SEM and XRD evidence, this study revealed the coupling mechanism between microstructural evolution (C–S–H formation and porosity reduction) and macroscopic dynamic response. This micro–macro linkage offers a new mechanistic explanation for the improved stiffness and damping characteristics.

Overall, the research establishes a new scientific basis for understanding the dynamic mechanical behavior of cementitious material-stabilized sludge soil and provides theoretical support for its resource utilization in transportation infrastructure.

## Figures and Tables

**Table 1:** Basic Physical Properties of the Soil.

$\omega_{L}$/%	$I_{L}$/%	$I_{P}$	$\rho_{d\max}$ $/g\cdot cm^{-3}$	ω/%
35.2	19.6	15.5	1.69	17.0

**Table 2:** Main Chemical Composition of Mineral-based Cementitious Material.

Chemical Composition	CaO	SiO_2_	Al_2_O_3_	Fe_2_O_3_	MgO	SO_3_	NaO	Others
Percentage	56.24	25.67	9.34	1.63	2.68	3.24	0.24	0.96

**Table 3:** Dynamic Triaxial Test Design.

Sample No.	f/Hz	c/%	t/d
X1-*f* 1-*c* 8-*t* 3	1	8	3
X2-*f* 2-*c* 8-*t* 3	2	8	3
X3-*f 3*-*c* 8-*t* 3	3	8	3
X4-*f* 2-*c* 3-*t* 3	2	3	3
X5-*f* 2-*c* 5-*t* 3	2	5	3
X6-*f* 2-*c* 8-*t*-3	2	8	3
X7-*f* 2-*c* 11-*t* 3	2	11	3
X8-*f* 2-*c* 8-*t* 1	2	8	1
X9-*f* 2-*c* 8-*t* 3	2	8	3
X10-*f* 2-*c* 8-*t* 7	2	8	7
X11-*f* 2-*c* 8-*t* 14	2	8	14
X12-*f* 2-*c* 8-*t* 28	2	8	28

## Data Availability

The original contributions presented in this study are included in the article. Further inquiries can be directed to the corresponding author.
